# Clinical Use of Skull Tap Vestibular Evoked Myogenic Potentials for the Diagnoses of the Cerebellopontine Angle Tumor Patients

**DOI:** 10.1155/2014/135457

**Published:** 2014-04-02

**Authors:** Erdem Yavuz, Magdalena Lachowska, Katarzyna Pierchała, Krzysztof Morawski, Kazimierz Niemczyk, Rafael E. Delgado

**Affiliations:** ^1^Department of Biomedical Engineering, McArthur Annex, University of Miami, Coral Gables, FL 33124, USA; ^2^Intelligent Hearing Systems Inc., 6860 SW 81st Street, Miami, FL 33143, USA; ^3^Department of Otolaryngology, Medical University of Warsaw, Ulica Banacha 1a, 02-097 Warsaw, Poland

## Abstract

*Objective.* To document our experiences using a new skull tapping induced Vestibular Evoked Myogenic Potentials (tap VEMPs) technique combined with standard Auditory Vestibular Evoked Myogenic Potentials (AC VEMPs) for advanced clinical assessment of cerebellopontine angle tumor (CPAT) patients. *Design and Study Sample.* Three patients were selected in order to highlight observations shown in a larger patient population and to show the variability of the findings. Both tap VEMPs and AC VEMPs were acquired from the sternocleidomastoid muscle (SCM) with EMG-based biofeedback and monitoring. *Results.* The usefulness of VEMPs was demonstrated, indicating the presence of a tumor and contributing additional information as to the involved nerve bundles in two out of the three cases. *Conclusion.* Due to the sensory organ dependency and related innervations differences, acquiring both AC VEMPs and tap VEMPs is likely to increase the probability of diagnosing CPATs and provide more information on the involved vestibular nerve bundles. This study demonstrates the feasibility of the possible expansion and combination of tap VEMPs and AC VEMPs techniques into a clinical diagnostic battery for advanced assessment of CPAT patients and its contribution as a guideline for the use of tap VEMPs in general.

## 1. Introduction

Vestibular Evoked Myogenic Potentials (VEMPs) are one of the most recent methods added to the vestibular organ test battery. Although the initial reports of the VEMPs can be dated back to the early 1935 [1], the current highly used method based on the myogenic activity recordings from the sternocleidomastoid (SCM) muscle was introduced by Colebatch et al. [2, 3]. Sound evoked cervical VEMPs rapidly gained the attention of both researchers and clinicians, resulting in numerous publications describing the clinical and research potential of VEMPs. Additional research extended the VEMP stimulation from auditory to skull tapping [4], bone conduction vibrations [5, 6], and galvanic stimulation [7, 8]. In addition to the SCM muscle, a variety of muscles were also shown to produce VEMPs, such as masseter [9], gastrocnemius, tibialis anterior, biceps femoris, quadriceps [10], and extraocular muscles [11, 12].

Auditory cervical approach is currently the most investigated VEMP acquisition method. The current consensus on the sensory organ responsible for the Auditory Cervical VEMPs (AC VEMPs) is the saccule [13, 14]. Saccular origin suggests that the inferior vestibular nerve functional integrity is essential in the generation of AC VEMPs as the majority of the sacculus innervation is carried by this nerve [15–18]. More recently, skull tapping VEMPs (tap VEMPs) have been heavily investigated by various authors. The tap VEMPs are proposed to generate a more complex stimulation paradigm described as containing two different mechanisms, one resulting in an ipsilateral inhibitory activity on the SCM and the second acting bilaterally producing a response with a polarity which is opposite for the two SCM muscles [19]. Although it is still not clear which part of the vestibular organ is responsible for the tap VEMPs, the utricle has been designated to be the origin of the unilateral and bilateral components [19–22]. However, the vibrations conducted via bone are propagated to a large region on the skull and may stimulate other parts of the vestibular organ. Therefore, skull tapping is a more complex stimulus type, likely to result in activation of multiple sensors on both sides of the head bilaterally activating the superior and inferior vestibular nerves [23, 24]. Evidence strongly suggests that tap VEMPs, to a significant extent, are dependent on superior bundle of vestibular nerve; they are more often affected in patients with vestibular neuritis (which usually affects only the superior vestibular nerve), in contrast to AC VEMP [25]. It has been also reported that the tap VEMPs can be recorded despite selective section of the inferior vestibular nerve [26], indicating that tap VEMPs strongly rely on the superior vestibular nerve [23].

Initial tap VEMP studies were performed by using a manually controlled reflex hammer attached to a triggering mechanism [4]. Skull tapping via reflex hammer is prone to deliver varying amount of momentum with each individual hit to the test subject, thus causing variability in the response. Later studies used mechanically controlled vibration and impact generating devices for skull tapping in order to standardize the amount of momentum delivered with each tap [23, 27, 28]. These devices are generally held by hand against the impact site on the skull. The forces delivered by these devices are also prone to variability as the position on the head and the hand position are likely to change during the recordings. The major source of variability in VEMP recordings is shown to be the contraction level of the muscle that is used as the electromyelography (EMG) source. It has been shown that the VEMP amplitude is directly related to the strength of the background SCM muscle activity and is absent at rest [3, 29, 30]. In general, during VEMP recordings, the subject is asked to push the head against a structure to ensure a certain amount of contraction in the cervical muscles [3] or asked to lie in supine position lifting their heads up [4] or asked to turn their heads away from the stimulus [31]. Although the abovementioned methods for muscle contraction can help to maintain muscle activity, none can be used to guarantee a constant EMG activity range. To overcome this problem, EMG-based biofeedback methods were developed to increase the cooperation of the subjects so that the subject can monitor and correct the contraction level of the muscle. In this approach, a muscle activity value is calculated from the rectified EMG signals and displayed to the subject as feedback [21, 32].

VEMPs stand out as a promising tool in clinical practice as they are noninvasive and easy to acquire with low time and instrumentation cost. Additionally, when acquired together AC VEMPs and tap VEMPs may be used as a tool to identify the functional integrity of inferior and superior vestibular nerves.

Despite large amount of research on VEMPs and constant development of the instrumentation and methods for the clinical usage, VEMPs and in particular tap VEMPs are still not well established clinically due to a number of issues. Some of these issues are lack of a standardized tap VEMP specific device, limited or no quantification on the delivered force and instantaneous EMG activity, lack of clinical experience, sensitivity, specifications, and also lack of established clinical protocol for the use of tap VEMPs.

In this paper, our main goal is to establish a clear, repeatable protocol for the clinical assessment of CPAT cases, geared by the recent developments in the objective vestibular functional evaluation methods, particularly the complimentary use of AC VEMPs and tap VEMPs in daily clinical practice. In our department VEMPs are part of a routine testing on various vestibular problems, particularly on patients with cerebellopontine angle tumors (CPAT). In this study, we present results of AC VEMP and tap VEMP recordings acquired with the use of a new prototype automated skull tapping device (Intelligent Hearing System Inc., Miami, FL) that can be stabilized on the skull using a headband that ensures a fixed placement and contact pressure. Additionally, an EMG standardization method integrated into the acquisition software is used to further minimize the variability of the AC VEMP and tap VEMP recordings. This paper describes our findings on 3-case examples from a growing patient data pool which will be presented in the future.

## 2. Material and Methods

### 2.1. Clinical Investigation Protocol

In our department, when a patient is identified as a potential CPAT case, our general protocol is to conduct a diagnostic battery on the patient by following a diagnostic procedure for vestibular or acoustic schwannoma [33] using the standard tests with the complimentary addition of AC VEMPs and tap VEMPs. The order of the tests in the following list reflects the escalation in the diagnosis towards a CPAT case.

1. Patient history.
2. Otoscopy.
3. Audiological assessment (particularly for signs of compression reflecting the involvement of other nerves that share the same trajectory in the auditory canal [34–36]) that involves pure tone audiometry, speech discrimination test, impedance audiometry with stapedius reflex, and auditory brainstem response (ABR).
4. Examination of the patients by acquiring auditory and skull tapping VEMPs according to the protocol that will be described in detail below. Due to the sensory organ dependency and related innervations differences, as mentioned above, recording both the AC VEMPs and tap VEMPs further increases the potential of identification of the affected nerve bundles. For instance, in cases where no or reduced AC VEMPs are observed but tap VEMPs are present, a nonfunctional inferior bundle but a functioning superior vestibular nerve can be estimated.
5. The magnetic resonance imaging (MRI) with gadolinium enhancement is currently viewed as the most accurate diagnostic tool for VIII nerve schwannoma capable of identifying tumors as small as 3 mm in size. On the other hand it is quite costly and it is not performed at the early stages of the diagnostics procedure. In many countries, where health services are rationed, a clear indication of tumor presence is necessary for scheduling an early MRI appointment. The MRI is performed if tests described above indicate the high suspicion of the CPAT according to cross-check rule. In MRI tumor size, location in the internal auditory canal (which part of the canal) and brainstem compression if present are described. This information is very helpful in consideration of surgery approach which depends on the size and location of the tumor and the degree of hearing loss.
6. Clinical and electrical examination of the facial nerve is performed to test the facial nerve involvement.
7. Surgical referral.

While the MRI provides information on tumor presence and its size and location in the internal auditory canal (which part of the canal) that are essential for the surgeon, it does not provide information about which nerve bundles are involved in the process. The information provided by ABR and AC VEMPS and tap VEMPs together is very useful for a surgeon due to the relationship of cochlear and vestibular nerves in the internal auditory canal. The vestibulocochlear nerve divides into individual nerves in the lateral aspect of the internal auditory canal: cochlear nerve more anteriorly and both vestibular nerves superior and inferior more posteriorly [34–36]. The facial nerve courses anterior like cochlear nerve but remains more superior to it. The nerves all together rotate 90 degrees in their course from the fundus of the internal auditory canal to the cerebellopontine angle, so that the cochlear nerve rotates from anterior to posterior but stays most inferior [34]. As the vestibulocochlear nerve divides into individual nerves, the presence of a tumor can be reflected by malfunctions of these nerves depending on which nerve the compression occurs.

### 2.2. Acquisition of AC VEMPs and tap VEMPs

All VEMP recordings were conducted using surface electrodes placed on the skin above SCM muscle. The positive electrodes were placed bilaterally on the midpoint of the SCM muscle measured between the points where the muscle was connected to the mastoid and the sternum. The negative electrodes were positioned on the sternum and the SCM junction. The ground electrode was placed laterally on the zygomatic bone so that the placement of the skull tapper on the forehead was possible for tap VEMP recordings.

All VEMPs were acquired using SmartEP evoked potential acquisition system on the IHS USB Box platform (Intelligent Hearing Systems, Inc., Miami, FL). Recordings were performed with sampling period of 400 ms with 5K amplification filtered using a 6 dB per octave band pass filter with highpass cutoff set at 30 Hz and lowpass cutoff set at 1500 Hz. The AC VEMPs were collected by averaging 128 sweeps and tap VEMPs were acquired averaging 64 sweeps. Less number of sweeps was used for the tap VEMPs as skull tapping was found to generally generate larger amplitude responses compared to the auditory counterpart. For each recording site and type two sets of recordings were made which were later averaged to increase the signal to noise ratio. The two sets of recordings were used to monitor the repeatability of the recorded signals. The AC VEMPs were evoked by 5000 ms long 500 Hz tone bursts conditioned by an exact Blackman window delivered via ER3a ear insert phones (Etymotic Research, Inc., Elk Grove Village, IL) at 100 dBnHL. The tap VEMPs were evoked by a prototype skull tapping device produced by Intelligent Hearing Systems Corp. The skull tapper was composed of an electromagnetic push type mechanism automatically controlled by the software to deliver a controlled force with a hit onset detection mechanism. The piston of the tapper was held at the same position for 100 ms following the hit at the surface interfacing the skull to ensure a unidirectional force delivery. The delivered force was measured to be 10.5 N using a commercially available artificial mastoid device (type 4930) Bruel & Kjær (Nærum, Denmark). Details on the skull tapper device will be described in a separate article. A stimulation rate of 3.1/s was used for both VEMP recording types. The recording system uses an EMG-based biofeedback monitoring method to minimize the variation in the SCM muscle contractions and thus the variation in the amplitude of VEMPs. This method is based on continuous monitoring of pre- and poststimulus EMG activity. In this method two conditions had to be fulfilled for a window of a recording cycle to be accepted into the average: (a) the root means squared (RMS) EMG activity had to fall into a range set by the user (generally minimum at 50 *μ*V and maximum at 150 *μ*V RMS), and (b) the poststimulus activity should not exceed a user set artifact rejection value. If both cases were satisfied an illustration of a smiling face was shown on the monitor to the patient (this would indicate to the patient that the SCM muscle contraction was sufficient for recording and that he/she should stay in this position to complete the set of recordings). In addition, a green bar showing the actual EMG RMS levels was also presented. When any of the conditions was not met the smiling face was replaced by an upset face and the EMG bar color was turned to red. A secondary feedback indicator box that contained a red and a green LED light was also present. Both indicators were used to increase the patient cooperation with the SCM muscle contraction and minimize the muscle fatigue.

The skull tapper was placed on the skull at three locations: (a) at the midline on the forehead, (b) behind the left ear on the mastoid process, and (c) behind the right ear on the mastoid process. The stabilization of the skull tapper was ensured using an adjustable head band.

The recordings were performed with the patients comfortably resting in a supine position and lifting the head up towards midline. The patients were directed to just lift the head, with no shoulder and abdominal muscle activity if possible. During all recordings a researcher was present at all times directing the patient to increase or decrease the lift of the head or the turn movement to stay in the selected RMS EMG levels using the biofeedback monitor for guidance.

Three different types of recordings were conducted with AC VEMPs (two repetitions each):

1. head lift stimulus delivered to the left ear (AHLL),
2. head lift stimulus delivered to the right ear (AHLR),
3. head lift stimulus delivered to both ears (AHLB).

Three different types of recordings were conducted for the skull tapping evoked VEMPs (two repetitions each) with skull tapper located at

1. forehead head lift both sides recorded (FHLB),
2. mastoid head lift skull tapper located at left (MHLL),
3. mastoid head lift skull tapper located at right (MHLR).

**Figure 1:**
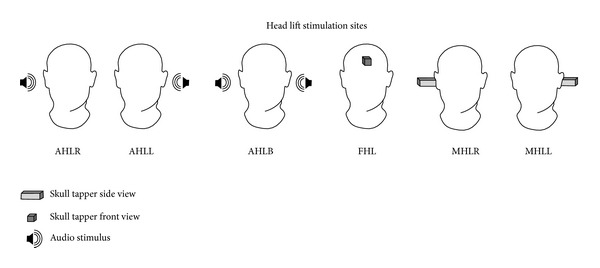
The stimulus presentations sites for the head lift recordings made for acoustic and skull tap VEMPs. Abbreviations in the illustration indicate the stimulus type and direction as follows: (1) head lift stimulus delivered to the right ear (AHLR); (2) head lift stimulus delivered to the left ear (AHLL); (3) head lift stimulus delivered to both ears (AHLB); (4) forehead head lift (FHL); (5) mastoid head lift skull tapper located at right (MHLR); (6) mastoid head lift skull tapper located at left (MHLL).

Therefore a total of 12 recordings were conducted for each patient (6 total types with two repetitions) before and after the surgical intervention as shown in Figure 1.

The recorded responses were normalized according to the prestimulus (base) EMG RMS calculations. Normalized values were used to assess the asymmetry ratios (AR) between the left and right side measurements. The usefulness of the normalization of AR measurements is still under debate [37]. The following equation (1) was used for the asymmetry ratio, where the  *A*_*L*_  is the amplitude measure from the left side recording between the first positive and first negative peak and similarly  *A*_*R*_  is the amplitude measured on the right side. Corrected AR of up to 35% is considered normal [38]:

(1) $$\begin{array}{c} \text{ Asymmetry Ratio}=100\left|\frac{\left(A_{L}-A_{R}\right)}{\left(A_{L}+A_{R}\right)}\right|. \end{array}$$

During the course of this study additional VEMP recordings were made where the SCM contraction was sustained via the head turn to the sides. In this paper we only present the results and the details of the head lift data as we found the head turn method to be unreliable when analyzed via asymmetry ratio approach. We prefer and advocate the head lift approach as it is much easier to control and identify if a patient's head is symmetrically lifted or not. For this reason we believe that the VEMPs recorded via head lift are less prone to be affected by the orientation differences of the vestibular organs during the recording sessions compared to head turn with the current instrumentation limits. Additional feedback monitoring of the 3D positioning of the head by using additional means such as gyroscopes and/or accelerometers could help to minimize this variability.

### 2.3. Participating Patients Details

*Patient Number 1*. Patient number 1 was a 43-year-old man who suffered from slight tinnitus and slight hearing loss in his right ear with no vestibular problems prior to admission. Audiological tests revealed a small high-frequency sensorineural hearing loss on the right side with pure tone average (PTA) for 3 and 4 kHz = 35 dBHL. The speech discrimination score at 60 dB was almost normal (80%, scored 100% at 70 dB). Impedance audiometry revealed normal middle ear function and normal stapedius reflex thresholds on both sides. Normal response to click stimulus at 90 dBnHL was present on the left side in ABR but on the right side ABR showed retrocochlear abnormality. Vestibular caloric test and videonystagmography showed normal responses on both sides with no central vestibular disorders. Details on the VEMP recordings will be described in the Results section. MRI revealed a tumor in the internal auditory canal; the tumor was 12 × 9 mm in size. Clinical and electrical examination (using electromyography) of the right facial nerve revealed its normal function.

Middle fossa approach was chosen for the surgery attempting to preserve the hearing. The tumor appeared to arise from cochlear nerve and was attached to other nerves in the internal auditory canal.

*Patient Number 2*. Patient number 2 was a 51-year-old woman. Prior to the admission, she complained about tinnitus and hearing loss in the right ear with some symptoms of right facial nerve paresis. Pure tone audiometry revealed sensorineural hearing loss on the right side with PTA for 0.5 and 1 kHz = 30 dBHL and for 2 and 4 kHz = 57.5 dBHL, and the speech discrimination score at 60 dB was 30% for the right ear. Normal middle ear function was proved with tympanometry but stapedius reflexes were missed. Normal response to click stimulus at 90 dBnHL was present on the left side in ABR and no reproducible waves on the right. Vestibular caloric test and videonystagmography revealed aflexia on the right side with no central vestibular abnormalities and normal function on the left. Clinical and electrical examination (electromyography) of the right facial nerve revealed its abnormal function. Details on the VEMP recordings will be described in the results section. MRI showed a 4 × 9 mm tumor in the right internal ear canal.

Middle fossa approach was selected to increase the possibility of hearing preservation. The tumor seemed to arise from superior vestibular nerve but involved the inferior one as well.

*Patient Number 3*. Patient number 3 was a 26-year-old woman whose main complaint was tinnitus on the left side. At the admission she stated not having any other hearing or vestibular problems. Auditory testing revealed normal hearing in both ears in all audiometric frequencies with the pure tone average for 0.5, 1, 2, and 4 kHz = 2.5 dB and speech discrimination score at 60 dB was 100%. Impedance audiometry revealed normal results. Auditory brainstem response showed normal reproducible waves on both sides. Vestibular caloric test and videonystagmography showed normal responses on both sides with no central vestibular disorders. Left facial nerve function was normal. VEMPs will be discussed in the Results section. Due to the patient's history of asymmetric tinnitus and young age it was decided to perform MRI. The MRI revealed a tumor in the internal auditory canal; the tumor was 7 × 5 × 15 mm in size.

For the surgery middle fossa approach was selected for the hearing preservation. The tumor seemed to arise from superior vestibular nerve.

This study is a part of a retrospective-prospective project that was approved by the Ethics Committee Review Board at the Medical University of Warsaw, where the VEMPs recordings have been conducted. The project conforms with The Code of Ethics of the World Medical Association (Declaration of Helsinki).

## 3. Results

**Figure 2:**
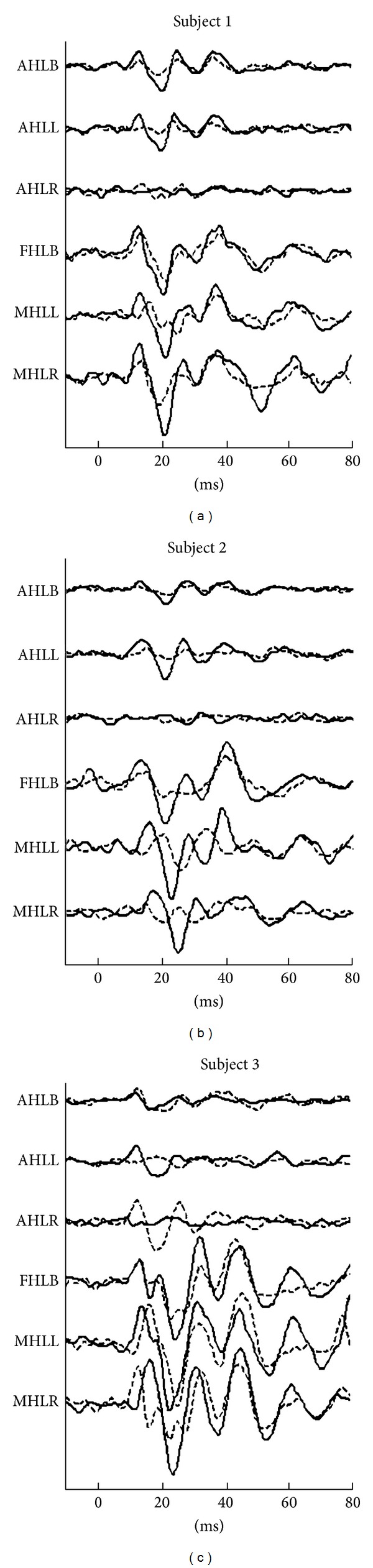
VEMP waveforms recorded from all 3 subjects. All the waveforms are normalized according to prestimulus EMG activity. In this figure left side recordings are plotted as solid lines while right side recordings are plotted as dashed lines. Abbreviations are used in this figure as follows. AHLB: auditory head lift both ear stimulation recorded bilaterally; AHLL: auditory head lift left ear stimulation recorded bilaterally; AHLR: auditory head lift right ear stimulation recorded bilaterally; FHLB: head lift recording tapper located at forehead recorded bilaterally; MHLL: head lift recording tapper located at left mastoid recorded bilaterally; MHLR: head lift recording tapper located at right mastoid recorded bilaterally.

**Figure 3:**
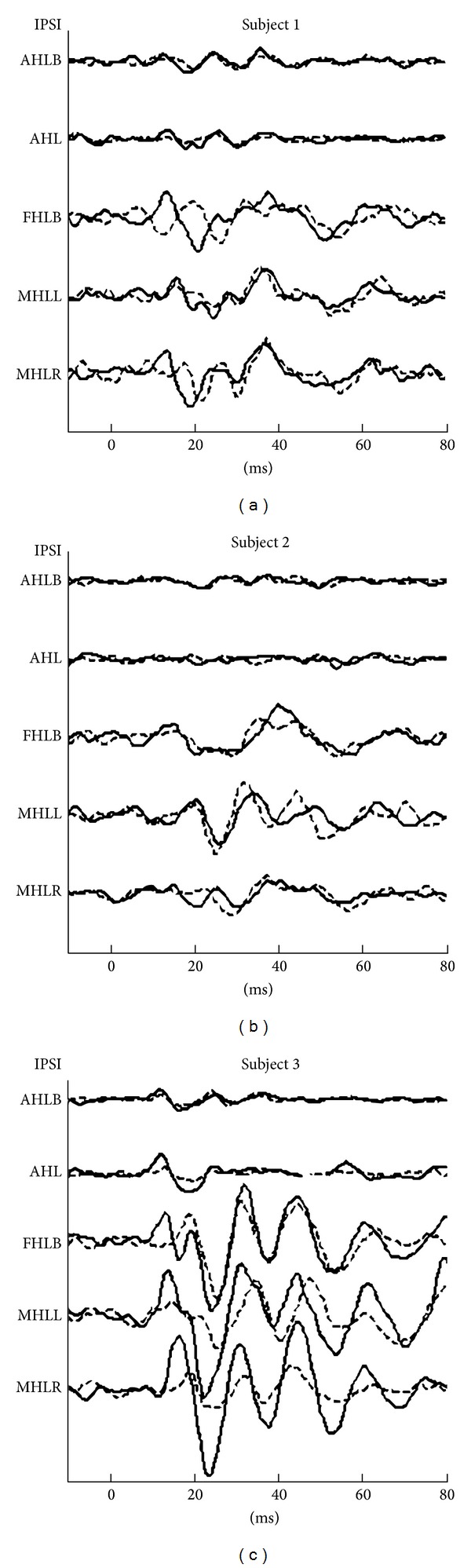
Preop (solid lines) and postop (dashed lines) VEMP waveforms recorded ipsilateral to the pathology from all 3 subjects. All the waveforms are normalized according to prestimulus EMG activity. Abbreviations are used in this figure as follows where ipsilateral indicates the location in return to the pathology location. AHLB: ipsilateral auditory head lift recording with both ears stimulated; AHL: ipsilateral auditory head lift recording in return to ipsilateral stimulation; FHLB: ipsilateral head lift recording tapper located at forehead; MHL: ipsilateral head lift recording tapper located at left mastoid recorded bilaterally; MHLR: head lift recording tapper located at ipsilateral mastoid.

Table 1 shows AC VEMP and tap VEMP results with values for P1 latencies and corrected amplitudes of the responses along with corrected asymmetry ratios.  Figure 2 presents the results of the preoperative (preop) recordings acquired by both VEMP techniques.  Figure 3 shows the change observed between the preop and postoperative (postop) responses recorded from the ipsilateral side to the tumor.

*Patient Number 1*. In this patient, highly large corrected asymmetry ratio (AR) values for AC VEMPs (AHLB AR = 47.59%; AHLL versus AHLR AR = 45.43%) were observed. Values lower than 35% are considered normal [38] and we used them as referral. In close inspection of the waveforms from the contralateral side, one can easily see that the left side stimulation produced much smaller activity on the right side, where the right side stimulation did not produce a similar activity on the left side (Figure 2).

The tap VEMPs were symmetric for the forehead (FHLB AR = 19.15%) and for the mastoid skull tapping results recorded from the ipsilateral side SCM (right MHLR versus left MHLL AR = 16.84%).

The postop responses were compared with the preop data (Figure 3). The AC VEMPs results showed that P1 and N1 disappeared when the ear ipsilateral to the tumor was stimulated. In the bilaterally stimulated recordings, we observed the preservation of these peaks to an extent. Considering the surgical cut of the ipsilateral inferior bundle, the preservation of the peaks suggested the contribution of the contralateral side. Forehead placement tap VEMPs showed a reversal in the recorded P1-N1 peak complex. In all of the above recordings the later waveforms observed between 25 and 60 ms range which are generally regarded as cochlear in origin [24, 39] did not display a major change.

*Patient Number 2*. Patient number 2 medical history, along with unilateral sensorineural hearing loss, poor speech recognition, ABR, VNG, and facial nerve EMG results, pointed to impaired function of VIII and VII nerves on the right side.

In AC VEMPs we observed drastic differences between left and right side responses (AHLB AR = 43.49%). The right side auditory stimulation failed to generate a response where the left side stimulation evoked a robust response (Figure 2).

Skull tapping the forehead and each mastoid resulted in large ARs (FHLB AR = 57.60%; MHLR versus MHLR AR = 61.32%). Upon visual inspection of ipsilateral responses, the initial parts of the waveform morphology (P1-N1 region) were different (Figure 2.).

In the postop preop comparison (Figure 3), there were no significant changes in any of the responses recorded ipsilateral to the tumor in neither of the VEMP techniques for P1 and N1 peaks, suggesting that both bundles had total functional loss before the surgery. Additionally, similar to the first patient, late waveforms did not display a significant change.

*Patient Number 3*. In patient number 3, all audiological VNG and VEMP tests were within normal limits. Due to patient's young age and history of tinnitus on the left side this suggested that there might be a retrocochlear problem.

No asymmetry was observed in the AC VEMPs (AHLB AR = 16.62%; AHLR versus AHLL AR = 19.95%) and tap VEMPs (FHLB AR = 17.18%; MHLR versus MHLL AR = 19.29%). The mastoid ipsilateral tap VEMP recordings showed high similarity. In this case, both AC VEMP and tap VEMP results failed to point to a tumor presence. The MRI showed a tumor in the left internal auditory canal (7 × 5 × 15 mm). During the surgical intervention, the tumor was identified as arising from the superior vestibular nerve.

In the postop versus preop comparison (Figure 3) of the head lift AC VEMP recordings, no significant change on the tumor side was found when both ears were stimulated. However, significant difference was shown when the stimulation was at the side of pathology. For the tap VEMP, P1 peak could not be identified in any of the recordings and N1 peak amplitudes were diminished in all recordings except for the forehead placement where N1 latency and amplitude were preserved. In general, more variability in latency and amplitude was also present for the later waves as well.

## 4. Discussion

In today's audiovestibular clinical practice a large number of tests are available to determine the functionality of the audiovestibular system. The selection and the use of these tests are mostly limited by their selectivity, practicality, and costs. The rapid development of VEMP related research and our recent experience with AC VEMPs and tap VEMPs have led us to use VEMPs as an integral part of our diagnostic battery. In our department we routinely use the cervical VEMPs due to the longer history; thus there is larger amount of accumulated research on cervical VEMPs. Currently we are also investigating the use of relatively newer approach of ocular VEMPs and adding it to our collected data set. Although the recent research on ocular VEMPs has been encouraging for clinical usage, the technique has to still be refined to be used as a routine test. The cervical VEMPs are easy to acquire and can be efficiently used with quantification methods for ongoing muscle activity monitoring such as the one used in this study where acceptance and rejection muscle activity regions are used. Our near feature plan is to include the ocular VEMPs recording in our routine diagnostic tests ensuring that the adaptation of the muscle activity quantification methods we use is properly utilized.

Our results showed that the VEMP recordings were not only capable of identifying the CPAT but were also able to contribute additional information of involved nerve bundles in first patient. The observed abnormal audiological findings strongly suggested the initial diagnosis of vestibular schwannoma. The MRI pointed to the presence of a tumor located in the right internal auditory canal. VEMP results also supported the presence of a tumor, additionally pointing to the inferior vestibular nerve involvement. The surgery report described the tumor to be arising from cochlear nerve and to be attached to the other nerves in the internal auditory canal. The cochlear nerve is located closer to the inferior bundle of the vestibular nerve [34–36] and compression on the inferior nerve therefore was affecting the AC VEMPs results but not altering the proper function of the superior vestibular nerve (AR in tap VEMPs within normal limits [38]).

For the second patient, the audiological results along with VNG and VEMP results strongly suggested the tumor presence in the internal auditory canal which was supported by the MRI findings. The surgeon described the tumor arising from superior vestibular nerve involving the inferior bundle as well. During the surgery, the tumor was found to be bigger than initially described by the MRI, which was performed a few months before. As routinely done prior to the surgery, the other tests, including VEMPs, were performed to decide on the surgical approach and determine the possibility for hearing preservation. In this case, the tumor affected the function of all the nerves in the internal auditory canal. Our conclusion from the AC VEMP and tap VEMP findings was the involvement of both the inferior and superior nerves. The VEMPs were successful in pointing out both the tumor presence and the involvement of both of the vestibular nerves. An interesting observation was that the left and right responses recorded during both right side and left side auditory stimulation were highly similar, suggesting that the whole response seen in AHLB might have been driven by the left ear.

The third patient was a particularly interesting case because all tests performed along with AC VEMPs and tap VEMPs proved to be not helpful in the diagnosis of the vestibular schwannoma. Although the tumor was arising from the superior vestibular nerve, the tap VEMP failed to indicate presence of the tumor similar to the rest of the clinical tools, suggesting a normal function. In this case only the MRI was sensitive enough to show the tumor. The MRI was performed due to the history of the patient, her young age, and our experience in vestibular schwannoma cases. The reason why the recorded tap VEMPs were not able to detect the pathology is a good question to investigate.

We observed that the mastoid placement of the skull tapper created additional challenges. It was extremely difficult to replicate the same conditions when the skull tapper was placed on a surface of the mastoid region. Due to the 3-dimensional shape of the mastoid area, it was very difficult to maintain a steady 3D relation between the head and the skull tapper hit axis (thus with the vestibule axis) especially when tapper is moved from one side to the other. As with the current setup there was no chance of monitoring the relation between the skull tapper hit direction and the vestibular organs and it was not possible to limit the introduced variability. In the light of these observations (although we acquired data with skull tapper positioned at mastoids on both sides and described the results of the responses acquired from the ipsilateral SMCs), we preferred to relay and advocate the usage of the forehead stimulation results due to the limits of currently available instrumentation. The directional information revealed by the mastoid placement is complex and has to be carefully analyzed. For general clinical implementation additional safeguards are required. An additional feedback mechanism describing the orientation relation between the tapper and gravity, such as a gyroscope, could be helpful in minimizing the variability and thus could be useful in producing equivalent directional force delivery to each side when skull tapper is moved from left to right.

## 5. Conclusions

In this paper we introduced our efforts in adding the AC VEMPs and tap VEMPs to expend the clinical diagnostic tests used for advanced assessment in CPAT patients, hoping that it will serve as a guideline for other clinicians interested in utilizing tap VEMPs.

The patients presented in this study were selected to point out different results we observed among a larger patient population. The findings from the larger population group will be presented in a more detailed follow-up subsequent paper. The main goal of this paper was to focus on the protocol used and point at the variability in the findings.

In two of three presented cases AC VEMPs and tap VEMPs together proved to be helpful in establishing the diagnosis of CPAT providing more information on the tumor affected bundle of vestibular nerve (superior or inferior or both). Performing only AC VEMPs or only tap VEMPs would have provided insufficient information about the tumor and the vestibular nerve bundle involvement. In the third case, VEMPs failed to identify the vestibular schwannoma, which was clearly shown in MRI scans. In this case, our medical intuition and expertise with CPAT patients prompted us to perform MRI. Due to the high cost of MRI, it is typically not performed at the early stages of the diagnostics procedure in many countries. Once other tests indicate the possible presence of the CPAT an MRI is conducted.

Providing AC VEMPs and tap VEMPs to the available test battery in the assessment of CPAT is likely to improve the diagnostic process by providing more information on the involved vestibular nerve bundles. In addition, this information might be used later during the surgery. In many cases the MRI is not performed very short before the surgery. Usually it is performed earlier during the diagnostic process. The surgery takes place sometime after. As mentioned before, the MRI is quite costly and usually is not performed again if not much time passed. However, the ABR, AC VEMPs, and tap VEMP, once performed in diagnostic proceeding, might be repeated short before the surgery to clarify the presence along with possible compression characteristics of the tumor on the vestibular and/or auditory nerves. This combined information, from earlier MRI scans and latest electrophysiological tests, serves the surgeons as a guideline in their surgical approach and during tumor removal. It provides very useful information for the surgeon in making a decision about additional intraoperative monitoring of hearing. In our department intraoperative monitoring is routinely used in every CPAT surgery and many other ear surgeries, but intraoperative hearing monitoring is not routinely performed in most of the other clinics. In addition, the details from electrophysiological tests about the involved nerve bundles are useful in patients counseling and informing them on more realistic possible outcomes of the surgery like hearing preservations possibilities and a risk of hearing loss during the surgery, a risk of facial nerve paresis, and vertigo symptoms after the surgery.

## Figures and Tables

**Table 1:** Auditory and skull tap VEMP head lift results.

Patient	Analyzed parameter	Head lift											
		Auditory						Tapping forehead			Tapping mastoids IPSI responses		
		AHLB		AR (%)	AHLL	AHLR	AR (%)	FHLB		AR (%)	MHLL	MHLR	AR (%)
		Response from left SCM	Response from right SCM		Response from left SCM	Response from right SCM		Response from left SCM	Response from right SCM		Response from left SCM	Response from right SCM	
1	P1 latency (ms)	13.20	13.40		13.40	14.20		13.20	14.40		14.00	14.40	
	Corr. amplitude	**25.56**	**9.08**	47.59*	**22.96**	**8.62**	45.43*	**36.24**	**24.59**	**19.15**	**33.67**	**23.97**	**16.84**

2	P1 latency (ms)	14.00	13.60		14.60	NR		14.40	15.20		17.20	15.60	
	Corr. amplitude	**13.14**	**5.18**	43.49*	**23.51**	**NR**		**33.22**	**8.94**	57.60*	**38.19**	**9.16**	61.32*

3	P1 latency (ms)	12.60	12.80		12.80	13.00		14.00	13.60		14.40	13.20	
	Corr. amplitude	**10.19**	**14.14**	**16.26**	**19.62**	**29.40**	**19.95**	**44.88**	**31.72**	**17.18**	**58.23**	**39.40**	**19.29**

AHLB: head lift stimulus delivered to both ears; AHLL: head lift stimulus delivered to the left ear; AHLR: head lift stimulus delivered to the right ear; FHLB: Forehead Head Lift skull tapper located at forehead; MHLL: Mastoid Head Lift skull tapper located at Left; MHLR: Mastoid Head Lift skull tapper located at Right; P1: positive peak; corr. amplitude: corrected amplitude; SCM: sternocleidomastoid muscle; AR: Asymmetry ratio; NR: no response. Abnormal results of corrected asymmetry ratios (AR% > 35%) and no response results (NR) are marked with ∗.

## Abbreviations

ABR: Auditory brainstem response
AS: Asymmetry ratio
CPAT: Cerebellopontine angle tumor
ECochG: Electrocochleography
EEG: Electroencephalography
EMG: Electromiography
MRI: Magnetic resonance imaging
PTA: Pure tone average
RMS: Root means squared
SCM: Sternocleidomastoid
VEMP: Vestibular Evoked Myogenic Potential
AC VEMP: Auditory Vestibular Evoked Myogenic Potential
tap VEMP: Skull tapping induced Vestibular Evoked Myogenic Potential.

### AC VEMPs Three Types of Recordings

AHLL: Head lift stimulus delivered to the left ear
AHLR: Head lift stimulus delivered to the right ear
AHLB: Head lift stimulus delivered to both ears.

### tap VEMPs Three Types of Recordings with Skull Tapper

FHLB: Forehead head lift both sides recorded
MHLL: Mastoid head lift skull tapper located at left
MHLR: Mastoid head lift skull tapper located at right.
